# The Role of Bovine Milk-Derived Exosomes in Human Health and Disease

**DOI:** 10.3390/molecules29245835

**Published:** 2024-12-11

**Authors:** Monika Jabłońska, Tomasz Sawicki, Justyna Żulewska, Katarzyna Staniewska, Adriana Łobacz, Katarzyna E. Przybyłowicz

**Affiliations:** 1 Department of Human Nutrition, Faculty of Food Sciences, University of Warmia and Mazury in Olsztyn, 45f Sloneczna, 10-718 Olsztyn, Poland; tomasz.sawicki@uwm.edu.pl (T.S.); katarzyna.przybylowicz@uwm.edu.pl (K.E.P.); 2 Department of Dairy Science and Quality Management, Faculty of Food Science, University of Warmia and Mazury in Olsztyn, Oczapowskiego 7, 10-719 Olsztyn, Poland; justyna.zulewska@uwm.edu.pl (J.Ż.); adriana.lobacz@uwm.edu.pl (A.Ł.); 3 Department of Commodity Science and Food Analysis, Faculty of Food Sciences, University of Warmia and Mazury in Olsztyn, Pl. Cieszynski 1, 10-726 Olsztyn, Poland; kasta@uwm.edu.pl

**Keywords:** bioactive compounds, bovine, disease treatment, milk, milk-derived extracellular vesicles, miRNA, therapeutic application

## Abstract

Bovine milk is widely recognized as one of the most valuable sources of nutrients such as proteins, fats, vitamins, and minerals that support the development and health of the body. In recent years, there has been increasing scientific interest in exosomes, the small membrane-bound vesicles found in milk. Through their content (e.g., microRNA), exosomes can influence gene expression and modulate key signaling pathways within target cells. Results from in vitro and in vivo studies have shown that bovine milk-derived exosomes can alleviate intestinal inflammation by regulating signaling pathways and positively influencing the composition of the gut microbiota. They also improve cognitive function and support nervous system regeneration. In addition, exosomes promote bone health by stimulating osteoblast formation and inhibiting bone resorption, helping to prevent osteoporosis. Studies have shown that exosomes have beneficial effects on skin health by promoting collagen production, protecting cells from oxidative stress, and delaying the ageing process. Bovine milk-derived exosomes are a promising tool for the treatment and prevention of a variety of diseases, particularly those related to inflammation and tissue regeneration. Although these results are promising, further studies are needed to fully understand the mechanisms of action and the potential clinical application of milk exosomes in the prevention and treatment of different diseases.

## 1. Introduction

Milk is one of the oldest and most important foods in the human diet. From birth, it provides newborn mammals with the nutrients they need to grow, including proteins, fats, carbohydrates, vitamins, and minerals [1]. In different cultures around the world, milk and its products, such as yoghurt, cheese, and butter, have long been an important part of the daily diet. It is estimated that more than six billion people worldwide consume milk and dairy products, the majority of whom live in developing countries [2]. In addition to providing nutrients, milk also serves as a vehicle for a variety of biologically active molecules with significant implications for human health. These include immunomodulatory proteins, growth factors, and a wide range of extracellular vesicles, including exosomes, which are now being recognized for their central role in intercellular communication and potential therapeutic applications [3,4,5].

Exosomes are membrane-bound nanoscale-sized vesicles that are released by almost all eukaryotic cells, including the bovine mammary gland [4,5]. These vesicles are formed inside cells by the fusion of the inner membrane of endosomes to form structures called multivesicular bodies. After fusion of the multivesicular bodies with the plasma membrane, the exosomes they contain are released into the extracellular space [6]. The double lipid layer of exosomes is enriched in lipid components such as cholesterol, ceramides, and sphingolipids, which are responsible for the stability of exosomes and their ability to interact with target cell membranes. Within the lipid bilayer, there are also specific membrane proteins, including tetraspanins (e.g., CD9, CD63, and CD81), which play a key role in the fusion of exosomes with the membranes of other cells. In addition, these proteins are molecular markers that allow the identification of exosomes in different experimental approaches [6,7].

Due to their small size and characteristic membrane structure, exosomes are able to carry a variety of bioactive molecules, making them effective transporters of information between cells [5]. They are an essential part of intercellular communication, and their therapeutic and diagnostic potential is attracting increasing research interest [8]. The exosome cargo is extremely diverse and includes, among others, nucleic acids. One of the most abundant nucleic acid species in exosomes is microRNA (miRNA) [6]. MicroRNAs are short, non-coding RNA molecules essential for regulating gene expression at the post-transcriptional level. They play a key role in a number of biological processes, such as cell proliferation, apoptosis, immune response, and metabolism [9]. Other types of RNA found in exosomes include messenger RNA, ribosomal RNA, long non-coding RNA, transfer RNA, small nuclear RNA, small nucleolar RNA, and piwi-interacting RNA, all of which influence various biological processes [10]. In addition to RNA, exosomes also contain DNA, lipids (cholesterol, phosphatidylcholine, phosphatidylserine, sphingomyelin, and ceramides), and proteins (transmembrane proteins, membrane-associated proteins, and the soluble proteins sequestered in their lumen) that can activate specific signaling pathways in recipient cells [11].

One of the key mechanisms of exosomes is their ability to deliver cargo to other cells [6,10]. Exosomes can interact with target cells in three main ways: (1) they can bind to the cell membrane of the target cell through receptor–ligand interactions, which can lead to the activation of specific signaling pathways; (2) they can be taken up by the cell through endocytosis, allowing their contents to be released inside the cell; and (3) they can bind directly to the cell membrane of the target cell, allowing their contents to be transferred directly into the cytoplasm of the cell [10,11,12].

Exosomes derived from bovine milk have a high biocompatibility, which makes them suitable for effective use in various medical fields [4]. Due to their natural origin from milk, which is part of the human diet, these structures are well tolerated by the body without significant immunological reactions [13]. The high stability of exosomes in different biological environments, such as the gastrointestinal tract, further strengthens their application potential. Milk-derived exosomes are able to survive in the harsh digestive environment, allowing them to penetrate the intestinal barrier and reach various tissues of the body [14].

The isolation of exosomes from bovine milk is a key step in enabling their study and potential therapeutic applications. There are many protocols in the literature describing the isolation of exosomes from bovine milk. The most commonly used methods include differential ultracentrifugation, serial filtration, sucrose gradient centrifugation, size exclusion chromatography, polymer-based exosome precipitation kits, and advanced techniques such as tangential flow filtration and particle purification chromatography [15,16]. Differential ultracentrifugation is currently considered the best method for exosome isolation, which has several advantages, including high sample capacity, yield of large amounts of exosomes, and low isolation costs [17,18]. However, the use of ultracentrifugation-based methods is hampered by the fact that exosomes produced by this method are often contaminated with aggregates containing the protein casein, which routinely co-sediment with exosome particles during the purification process [15]. The recently published protocol of Weiskirchen et al. [16] incorporated chelating agents such as EDTA to mitigate this problem by breaking down casein aggregates and increasing exosome purity. These advances not only improved exosome isolate purity but also increased the reliability of downstream analyses such as exosomal RNA, protein, and lipid cargo profiling by minimizing non-vesicular contamination. Other isolation approaches have also been explored to overcome the limitations of ultracentrifugation. Ko et al. [19] demonstrated the feasibility of using oscillation-assisted tangential flow electrophoretic filtration with antifouling protection of micro-ultrafiltration membrane filters to achieve high-quality purification of bovine milk exosomes. This method reduces contamination and increases throughput but requires specialized equipment, which limits its scalability. Kaddour et al. [20], in turn, presented an efficient method for the purification of exosomes from bovine milk using size-controlled turbidimetry particle purification methods with high-resolution liquid chromatography, which combines a gradient exclusion column, fraction collection and online UV–vis/turbidimetry absorbance calculations. Although highly effective, this method requires extensive resources and expertise, making it less accessible for routine applications. The choice of isolation method plays a critical role in determining the integrity and biological activity of exosomal cargo. Ultracentrifugation, while effective in isolating large quantities of exosomes, often subjects the vesicles to mechanical stress, which can compromise both their structural integrity and the stability of their RNA, protein, and lipid cargo [21]. In contrast, size-exclusion chromatography and immunoaffinity capture methods are known for preserving the integrity of exosomal cargo due to their gentle processing conditions. However, these methods typically yield smaller quantities of exosomes [21]. Precipitation-based kits provide simplicity and ease of use but often lead to high levels of contaminants, such as proteins and polymer residues, which can interfere with cargo analysis and reduce the reliability of functional studies [21]. Future research should focus on developing standardized, scalable isolation protocols that combine the advantages of multiple techniques, such as ultracentrifugation and chromatography, to improve the reproducibility and purity of exosome isolates.

Exosomes offer significant potential for advancing our understanding of intercellular communication and developing innovative therapeutic strategies. This paper is a narrative review aimed at providing a comprehensive overview of the current knowledge on bovine milk-derived exosomes, focusing on their mechanisms of action in various pathological conditions and physiological processes. To ensure a robust selection of relevant literature, we conducted a structured search across academic databases (PubMed, Web of Science, and Scopus), using specific keywords related to bovine milk-derived exosomes and their effects on human health. We prioritized peer-reviewed journal articles published within the last decade that investigated the biological properties of bovine milk-derived exosomes, particularly their composition, mechanisms of action, and potential therapeutic applications. Our approach offers a narrative synthesis of the literature, incorporating findings from both in vitro and in vivo studies as well as systematic reviews.

## 2. The Impact of Exosomes on Intestinal Health and Function

Exosomes derived from bovine milk show significant potential in modulating intestinal health, acting in a multifaceted manner on intestinal barrier function, microbiota, and regulation of the inflammatory response. One study conducted on Mdr1a^−/−^ mice, a model of inflammatory bowel disease (IBD), showed that animals fed a diet depleted in bovine milk exosomes (ERD) had more severe disease symptoms when compared to mice fed a diet rich in exosomes (ERS) [22]. The exosomes were administered orally through the mice’s diet. Notably, stroma collapse, glandular hyperplasia, and additive microscopic disease scores were reduced by 56.7%, 23.5%, and 29.6%, respectively, in the ceca of ERS-fed mice compared to those of ERD-fed mice. Bovine milk-derived exosomes contain numerous miRNAs, including miR-200a-3p, which plays a key role in regulating the inflammatory response [23]. In the ERD diet, miR-200a-3p levels were significantly reduced (by ≤63% in the liver and ceca), which resulted in increased expression (by 35%) of the chemokine CXCL9 in serum. This chemokine plays a key role in the recruitment of inflammatory cells to the intestines, which results in an increased inflammatory response and, consequently, more pronounced histopathological changes in the intestine, such as glandular hyperplasia and stromal collapse [24]. In contrast, in mice fed the ERS diet, exosomal miR-200a-3p bound to CXCL9 mRNA, suppressed its translation, and reduced chemokine production, thereby preventing these pathological changes. The results of the study suggest that miR-200a-3p plays an important role in modulating the inflammatory response in IBD, and its deficiency may contribute to disease exacerbation [22].

In a mouse model of ulcerative colitis, bovine milk exosomes were shown to modulate the immune response by inhibiting the TLR4-NF-κB pathway and NLRP3 inflammasome activation [25]. The exosomes resuspended in phosphate-buffered saline (PBS) were administered orally through daily gavage, and colitis was induced with 3% dextran sulfate sodium (DSS). Bovine milk-derived exosomes significantly reduced the expression of key components of the TLR4-NF-κB signaling pathway, including TLR4, Myd88, COX2, phosphorylated IκBα, and p65 proteins. Similarly, the increase in the expression of the NLRP3 signaling pathway’s key components, namely NLRP3, apoptosis-associated speck-like protein, and pro-caspase-1, was significantly attenuated upon treatment of the mice with bovine exosomes. This comprehensive suppression of both pathways improved the Treg-Th17 balance in the gut, reducing inflammatory cell infiltration and tissue fibrosis [25].

In the study by Mun et al. [26], mice with DSS-induced colitis were used to investigate the effects of bovine milk-derived exosomes containing miRNA let-7a-5p. The exosomes were isolated from colostrum using ultracentrifugation, resuspended in PBS, and administered orally via gavage to mice. The results of the study showed that miRNA let-7a-5p downregulates the expression of *CDK6*, a gene critical for cell cycle control. Overexpression of *CDK6* is associated with abnormal cell growth, which can lead to inflammation [27]. By modulating *CDK6* expression in mice with colitis, exosomes may help restore normal cell proliferation, which is critical for alleviating the inflammatory response. Mun et al. [26] also demonstrated that supplementation with bovine milk-derived exosomes promoted the microbial abundance of *Akkermansia* (known to support the intestinal barrier and reduce inflammation) and β-hydroxybutyrate (a short-chain fatty acid with potent anti-inflammatory properties, which helps reduce inflammatory processes and promote intestinal tissue repair processes) [28,29].

The results of the study by Du et al. [30], also conducted in a mouse model of DSS-induced colitis, showed that exosomes from bovine milk significantly affect lipid and amino acid metabolism in the intestine. The exosomes were isolated from bovine milk using ultracentrifugation, resuspended in PBS, and administered orally to animals. Metabolomic profiling revealed that exosomes supplementation modulated metabolite levels disrupted by inflammation. Specifically, exosome treatment increased levels of eicosapentaenoic acid and docosahexanoic acid (lipid metabolites), which are known for their role in reducing inflammation [31,32]. In addition, a decrease in the levels of L-serine and L-glutamate was observed, suggesting a reduction in the catabolic processes typically associated with the metabolic shifts seen in active inflammation [33].

Milk exosomes also have the ability to protect and restore the integrity of the intestinal barrier. The study in mouse models of malnutrition showed that the oral administration of bovine milk-derived exosomes to mice on a low-protein diet resulted in increased intestinal cell proliferation and improved intestinal villus architecture (villus height increased to 260 nm compared to 223 in untreated mice, restoring 94% of control height), which contributed to epithelial regeneration and restoration of intestinal barrier integrity [34]. The beneficial effect of exosomes was associated with the activation of the Wnt signaling pathway, which upregulated *Lgr5* gene expression, thereby stimulating stem cell activity in intestinal crypts and facilitating tissue repair. Additionally, the administration of milk exosomes significantly increased the expression of claudin-3, a key tight junction protein in the intestinal epithelium, which further strengthened the intestinal barrier and its protective functions [35].

The results of the study by Tong et al. [36] revealed that bovine milk-derived exosomes significantly improved the integrity of epithelial tight junctions in both in vitro (intestinal epithelial cells) and in vivo (mouse) models. Exosome administration upregulated the expression of key tight junction proteins, i.e., ZO-1 and Occludin, which helped maintain intestinal barrier integrity [37]. Administration of exosomes also decreased levels of proinflammatory cytokines (e.g., IL-6 and TNF-α) and increased the levels of anti-inflammatory cytokine IL-10 in both models. The beneficial effects of bovine milk-derived exosomes were linked to the AMPK pathway [36].

Beyond the immune and structural benefits, there is growing evidence that bovine milk-derived exosomes may also influence the composition of the gut microbiota. As shown in the study by Tong et al. [38], oral administration of bovine milk-derived exosomes to mice increased the abundance of beneficial bacteria such as *Akkermansia*, *Muribaculum*, and *Turicibacter* while decreasing the levels of *Desulfovibrio*. *Akkermansia* is known to improve gut barrier function by supporting mucus production, *Muribaculum* contributes to carbohydrate metabolism, and *Turicibacter* plays a role in immune regulation [28,39,40]. The reduction in *Desulfovibrio*, bacteria that produce harmful hydrogen sulfide, helps to protect the intestinal epithelium from damage [41]. These changes in microbiota composition were correlated with a significant increase in the expression of genes important for gut barrier function and immune regulation. For example, the expression of *Muc2* (encoding mucin that protects the intestinal epithelium [42]) was upregulated by approximately 35% in the exosomes-treated mice compared to controls. Similarly, *RegIIIγ* (responsible for the production of antibacterial proteins [43]) and *Myd88* (which regulates the immune response via the Toll-like receptor pathway [44]) showed consistent upregulation in animals treated with bovine milk-derived exosomes [38]. According to Du et al. [45], the supplementation with bovine milk-derived exosomes led to enrichment in *Bifidobacterium*, *Dubosiella*, and *Lachnoclostridium* in mice. Exosomes were isolated from milk, resuspended in PBS, and administered to the animals orally by gavage. Treatment with exosomes was associated with an increase in the production of short-chain fatty acids, including butyrate and acetate, which are essential for maintaining intestinal homeostasis and suppressing the inflammatory response [45,46]. Research led by Li et al. [47] investigated the effects of bovine milk-derived exosomes on the gut microbiota of mice with DSS-induced colitis. The authors found that oral exosome administration helped preserve microbiota diversity as indicated by the Shannon index, which was significantly reduced in the DSS group when compared to control animals but restored to near-control levels in the exosome-treated mice. Exosome treatment also increased the relative abundance of bacteria from the *Lachnospiraceae* family and reduced the negative effect of DSS on bacteria from the *Roseburia genus*, which are crucial for the production of butyrate, important for maintaining gut health [47].

In summary, a growing body of evidence has demonstrated that bovine milk-derived exosomes play a key role in modulating gut health through a variety of molecular mechanisms and interactions with the microbiota. Exosomes act by modulating the inflammatory response, inhibiting key signaling pathways (e.g., TLR4-NF-κB), and activating repair mechanisms in the intestinal epithelium (e.g., the Wnt pathway). Notably, miRNAs within these exosomes, such as miR-200a-3p and let-7a-5p, regulate the expression of critical genes that improve intestinal barrier integrity and immune homeostasis, such as *CXCL9*, *CDK6*, or *Muc2*. Milk exosomes also have a significant impact on the gut microbiota, promoting the growth of beneficial bacteria like *Akkermansia* and *Bifidobacterium* while limiting the development of pathogens.

## 3. The Impact of Exosomes on Nervous System Function and Development

Exosomes derived from bovine milk have emerged as important regulators of nervous system development, as evidenced by studies investigating their effects on cognitive, neuronal, and sensorimotor functions in mice. In the study of Mutai et al. [48], adult mice were fed either an exosome-rich diet (ERS) or an exosome-depleted diet (ERD). The ERS diet contained normal levels of exogenous exosomes from milk, while the ERD diet lacked these vesicles. Mice on the ERD diet showed a significant decline in learning and spatial memory, particularly in younger animals. For instance, the time taken to locate the escape hole in the Barnes maze increased by up to 130% in ERD-fed mice compared to ERS-fed animals. Similarly, in the Morris water maze, mice on the ERD diet took significantly longer to locate and reach the submerged escape platform than animals fed the ERS diet. In addition, ERD-fed mice showed a significantly reduced response to sensory stimuli, as evidenced by problems with the filtration of acoustic stimuli. Analysis of gene expression in the tissue of the hippocampus revealed a downregulation of genes related to neuronal signaling, including those involved in axon guidance and calcium signaling, which likely accounts for the observed cognitive deficits [48].

Further supporting the role of milk exosomes in cognitive health, Zhou et al. [49] found that mice on the ERS diet performed significantly better on learning and spatial memory tasks, as assessed by the Barnes maze, than those on the ERD diet. Mice on the ERD diet scored nine times lower in the Barnes maze than ERS-fed animals. These mice also showed significantly reduced dendritic complexity in hippocampal neurons, suggesting reduced neuronal development. In addition to impaired cognition, ERD-fed mice were significantly more prone to kainic acid-induced seizures, with seizure severity scores being five times higher than those of mice on the ERS diet [49]. Another experiment conducted by the same authors examined the offspring of mothers fed either the ERS or ERD diet [49]. The offspring of mothers fed the ERS diet had better learning and memory skills and more developed dendrites in the hippocampus when compared to the offspring of mothers fed the ERD diet. Young mice had better results in the Barnes maze test and greater neuronal complexity in the hippocampal region, suggesting a beneficial effect of milk exosomes on brain development. In contrast, the offspring of ERD-fed mothers showed significant deficits in cognitive abilities, including challenges in learning and memory. They also had reduced dendritic complexity in the hippocampus and an increased susceptibility to epileptic seizures [49]. These finding showed that milk exosomes play a key role in neuronal development and improved cognitive function in both adult mice and their offspring. Due to the fact that microRNA sequences in exosomes are highly conserved across mammals, it can be assumed that similar effects may occur in different species, including humans [50]. The authors of the study pointed out that human milk contains higher concentrations of exosomes and microRNAs than infant formula [49]. This may have important implications for infant neurodevelopment, suggesting that enriching infant formula with exosomes may offer benefits. Additional studies in this field area may help to develop future recommendations regarding infant nutrition and early neurodevelopmental interventions.

## 4. The Impact of Exosomes on Bone Health and Regeneration

Osteoporosis, characterized by loss of bone mass and structural deterioration, is one of the most common metabolic bone diseases, particularly prevalent in the aging population. Its pathogenesis involves an imbalance between two cell types: osteoblasts, which mediate bone formation, and osteoclasts, which are responsible for bone resorption [51]. In recent years, bovine-milk-derived exosomes have been shown to have beneficial effects on bone health.

The results of the in vitro studies indicated that bovine milk-derived exosomes supported the differentiation of mesenchymal stem cells into osteoblasts [52], promoted osteoblast proliferation, and accelerated osteoblastogenesis [53]. These promising results were further confirmed by in vivo studies. In a mouse model of glucocorticoid-induced osteoporosis, oral administration of bovine colostrum-derived exosomes prevented bone loss and preserved the microstructure of trabecular bone in the femur [54]. Exosome treatment resulted in a significant increase in both bone mineral density and bone volume percentage compared to the control group, reversing the effects of glucocorticoid-induced osteoporosis. In addition, a beneficial effect of exosomes on the gut microbiota was observed, which may indicate the existence of a gut–bone axis affecting bone metabolism. In particular, *Lactobacillus* and *Bacteroides*, which were both significantly reduced in the osteoporosis model group, were restored to levels similar to those observed in controls in animals treated with exosomes [54].

In another study using an ovariectomy-induced mouse model of osteoporosis, bovine milk-derived exosomes also showed protective effects on bone tissue [55]. Exosomes were administered orally to the animals and effectively inhibited osteoclast differentiation, improved bone microstructure, and restored the levels of osteoporosis biomarkers. Microstructural improvements included increased trabecular thickness and bone mineral density in exosome-treated mice compared to untreated animals with osteoporosis. Exosome administration also improved intestinal barrier function and modulated the gut microbiota, particularly by increasing the abundance of *Bacteroides* associated with the production of short-chain fatty acids, including acetic and propionic acids. These short-chain fatty acids play a critical role in maintaining intestinal homeostasis and supporting bone health [56]. Correlation analysis revealed a strong association between changes in gut microbiota and short-chain fatty acid levels, with reductions in osteoporotic biomarkers. Moreover, exosome treatment significantly reduced levels of pro-inflammatory cytokines TNF-α and IL-17 while increasing the anti-inflammatory IL-10, thereby reducing inflammation and promoting bone repair [55].

In another study conducted in a mouse model of osteoporosis induced by ovariectomy, oral administration of exosomes to animals improved femoral stiffness and microstructure, reduced the number of osteoclasts, and decreased the RANKL/OPG ratio both in serum and in bone [57]. The RANKL/OPG ratio is a key indicator of the balance between bone resorption and formation, as RANKL stimulates osteoclast differentiation and enhances bone degradation, while OPG acts as its natural inhibitor, preventing osteoclast activation [51]. In addition, Oliveira et al. [57] showed that exosomes increased the number of osteocytes and osteoblasts, suggesting their beneficial effect on bone regeneration in estrogen-deficient mice. Interestingly, the beneficial effects of exosomes on bone health was also demonstrated in mice with obesity induced by a high-carbohydrate diet. Obese mice showed signs of bone loss, and exosome administration reversed these changes, improving bone health. Furthermore, mice on a high-carbohydrate diet had elevated RANKL levels and RANKL/OPG ratio. Exosome treatment restored these bone parameters to near-control levels. Although body weight and cholesterol levels were not changed, administration of exosomes reduced serum glucose and triglyceride levels, suggesting a beneficial effect on metabolism [57].

In summary, the results of the study show that exosomes derived from bovine milk have significant effects on bone health, protecting against bone loss and modulating cellular and molecular processes related to bone metabolism. This effect may be related to both direct effects on bone cells and indirect regulation via the gut–bone axis. Although these results are promising, further studies are needed to fully understand the mechanisms of action and the potential clinical application of milk exosomes in the prevention and treatment of osteoporosis.

## 5. The Impact of Exosomes on Skeletal Muscle Function

Exosomes derived from bovine milk have also emerged as a potential modulator of the metabolism and function of the muscle. While these extracellular vesicles have shown promise in promoting protein synthesis and cell growth in vitro, their effects in vivo are less consistent. The study by Mobley et al. [58] demonstrated that exosomes derived from bovine whey protein increased muscle protein synthesis by 45% compared to control cells. Exosome treatment also increased myotube diameters (by 29%), and this effect was associated with modulation of the expression of genes critical for protein synthesis (PTEN and eIF4A) and an increased (by 35%) in bovine-specific miRNAs, namely miR-214.

In contrast, the results of the in vivo study by Leiferman et al. [59] demonstrated that no significant differences in muscle amino acid levels or grip strength were observed between mice fed a diet with RNA-containing exosomes (ERS) and an RNA-depleted diet (ERD). In addition, amino acid levels in skeletal muscle were unaffected by the diets, as confirmed by principal component analysis. Gene expression analysis revealed only minor differences in the expression of several genes. For example, the expression level of *Tmem100* was approximately 2.5-fold higher in the EDR group compared to the ERD group, while the expression levels of *Rhobtb1* and *Socs2* were 2.22-fold and 2.86-fold lower, respectively. The results suggest that most milk exosomes and their RNA may accumulate in tissues other than skeletal muscle. The authors speculated that more pronounced effects might be observed in pathological conditions such as sarcopenia, which is a potential direction for future research [59].

The results of a study on the effect of exosomes derived from bovine milk on rat skeletal muscle showed that an exosome-depleted diet resulted in an unexpected anabolic effect, as evidenced by an increase in muscle fiber cross-sectional area (by 12%) and a 20% increase in total RNA levels [60]. Although the exosome-depleted diet was expected to have a negative effect on markers of anabolism, the observed changes included an increase in the expression of genes related to cell adhesion, fatty acid biosynthesis, and protein kinase activity. In addition, the exosome-depleted diet reduced the emission of reactive oxygen species in muscle (by 25%) and increased levels of the antioxidant protein GPX (by 30%), which suggests a reduction in oxidative stress [60]. Both studies provide interesting, albeit contrasting perspectives on the role of milk exosomes in muscle function. Leiferman et al. [59] suggested a moderate effect of an exosomes-rich diet on muscle regeneration and protein translation, with no clear effect on muscle performance. Parry et al. [60], in turn, showed that the exosome-depleted diet can paradoxically promote muscle growth and reduce oxidative stress, suggesting more complex mechanisms that regulate muscle function in response to dietary modifications. These studies highlight the need for further analysis to better understand how dietary modification of exosomes affects skeletal muscle function at the molecular and physiological levels.

## 6. The Impact of Exosomes on Skin Regeneration and Hair Growth

Bovine milk-derived exosomes have received increasing attention as a potential therapeutic agent, including in the area of skin and hair care. Exosomes from bovine colostrum have been shown to have great repair potential, particularly in mitigating the effects of skin ageing and repairing damage caused by UV radiation [61]. The results of the in vitro study conducted by Han et al. [61] showed that colostrum-derived exosomes promoted the proliferation of fibroblasts by 136% under normal conditions and by 109% under UV-C irradiation. This effect was mediated, in part, by activating the Wnt/β-catenin signaling pathway, which led to increased production of collagen, a key protein responsible for skin elasticity and strength, directly resulting in improved skin structure [62]. The results of the study also showed that exosomes reduced (by 56.1%) the expression level of genes encoding metalloproteinases enzymes that break down extracellular matrix proteins. Metalloproteinases play an important role in tissue remodeling, but their excessive activity can lead to premature skin ageing [63]. Therefore, modulation of metalloproteinase activity by exosomes is critical for skin regeneration and prevention of degradation. In addition, exosomes significantly reduced intracellular oxidative stress in keratinocytes exposed to UV-C radiation. This reduction was achieved via the glutathione pathway, as indicated by increased levels of glutathionylated proteins. Protection against oxidative stress is an important element in anti-aging since reactive oxygen species cause the degradation of skin structures such as collagen [61].

Exosomes derived from bovine milk also demonstrate the potential to improve skin hydration and reduce wrinkles [64]. In the study, exosomes were directly taken up by keratinocytes and fibroblasts, where they modulated the expression of key genes involved in skin moisture regulation and structural integrity. In keratinocytes, exosome treatment increased the expression of filaggrin by approximately three times and CD44 (the receptor for hyaluronic acid) by over 60%. Filaggrin plays a key role in maintaining the skin barrier by converting into amino acids that help the skin retain water [65]. CD44 facilitates the uptake of hyaluronic acid, which contributes to skin hydration [66]. Stimulating the expression of these proteins can lead to more effective water retention and strengthening of the skin barrier. In fibroblasts, exosome treatment increased the expression of HAS2, an enzyme responsible for hyaluronic acid synthesis [67], by more than two times. In addition, exosomes mitigated the reduction of collagen types I and III in UV-exposed fibroblasts, further supporting skin regeneration [64]. These promising results have led to clinical trials to assess the efficacy of exosomes in improving human skin conditions. The studies involved 31 women aged 26 to 45 years. Exosomes were diluted with water to a concentration of 60 μg/mL and applied to the facial skin in the morning and evening for 28 days. Exosomes were shown to increase skin hydration by 5.6%, especially in the group of older participants, and reduce the number of wrinkles by 4.99%. Moreover, exosomes improved skin elasticity and reduced wrinkle area, which indicates their anti-aging potential [64]. In this study, the safety of exosomes in terms of allergies and potential skin irritation was also tested in both animals and humans. In animal studies, exosomes were tested for skin allergy, photo-allergy, recurrent irritation and photo-irritation. None of these tests showed allergic reactions, skin sensitization, or signs of irritation. In a follow-up study of 31 volunteers, patch tests at 0.5, 24, and 48 h showed no adverse skin reactions. Taken together, these results confirm the lack of sensitizing or irritating potential of exosomes when applied to the skin [64]. To the best of our knowledge, this is the only study to directly assess the safety of bovine milk-derived exosomes in relation to skin allergy and irritation. However, further studies are needed to generalize these findings and to address other potential allergic reactions associated with exosomes derived from bovine milk.

An intriguing aspect of bovine milk-derived exosomes is their ability to regulate melanogenesis, the process responsible for melanin production and skin pigmentation. The in vitro study on mouse melanoma cells and human melanocytes demonstrated that microRNA-2478, present in bovine milk exosomes, inhibits the expression of the *Rap1a*, which is crucial for melanin production [68]. Rap1a is a protein belonging to the family of small GTPases that acts as a regulator in various signaling pathways, including processes related to cell proliferation, differentiation, and migration [69]. Suppression of Rap1a disrupts the PI3K/Akt pathway, resulting in decreased tyrosinase activity and reduced melanin production, potentially leading to skin whitening [68]. Tyrosinase activity in mouse myeloma cells was reduced by 43% and 59%, and melanin synthesis decreased by 45% and 55% at 20 µg/mL and 50 µg/mL exosome concentrations, respectively. In human melanocytes, tyrosinase activity decreased by 34% and 66%, while melanin production decreased by 36% and 62% at the same exosome concentrations. These findings suggest that bovine milk-derived exosomes may become an important component of cosmeceuticals, especially those with skin-whitening effects, due to their low cytotoxicity and high biocompatibility [68].

Furthermore, the results of the study on the bovine milk-derived exosomes also showed their significant potential in promoting hair growth [70]. The in vitro study on human dermal papilla (DP) cells, which regulate the hair follicle cycle, showed that colostrum-derived exosomes significantly enhanced DP cell proliferation in a concentration-dependent manner. Importantly, these exosomes were also able to override the inhibitory effect of dihydrotestosterone (the primary cause of androgenetic alopecia) on DP cell proliferation, suggesting their potential for treating hair loss.

**Figure 1:**
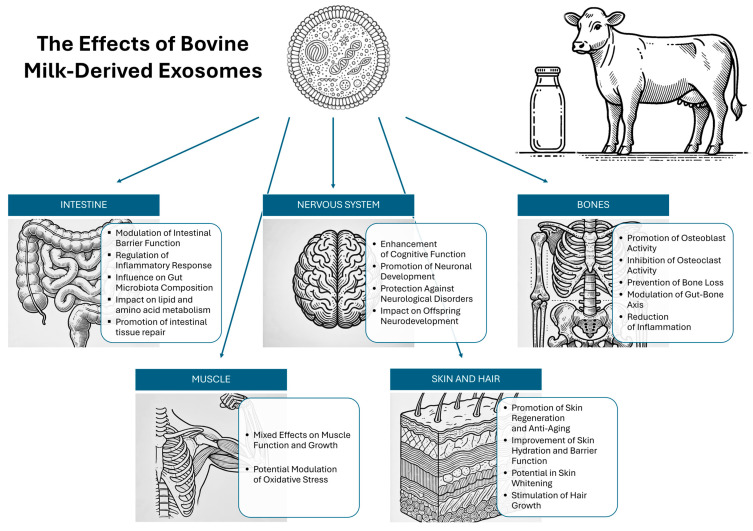
The effects of bovine milk-derived exosomes on human health and diseases. The graphics presented in this figure were created with the assistance of ChatGPT4.

The study in mice showed that exosome administration led to notable hair regeneration, with approximately 50% hair coverage achieved within 13 days when compared to the effects of minoxidil, a widely used anti-alopecia drug [71]. Histological analysis demonstrated that exosomes accelerated the transition of hair follicles from telogen (resting) to anagen (growth) phase, primarily through the activation of the Wnt/β-catenin signaling pathway, which was upregulated by increased expression of β-catenin (1.5-fold) and Wnt3a, stimulating DP cell proliferation and supporting hair regeneration. Unlike minoxidil, which can cause local skin irritation, exosomes did not induce inflammation, highlighting their potential as a safer therapeutic alternative. In addition, lyophilized exosomes retained their regenerative properties, which confirms their commercial potential and the possibility of long-term storage [70]. These results indicate the promising role of bovine milk-derived exosomes in hair regeneration therapies, opening up new possibilities for treating alopecia in a safe and effective manner. The above-mentioned effects of bovine milk-derived exosomes are summarized in Figure 1.

## 7. Additional Roles of Bovine Milk-Derived Exosomes

### 7.1. The Role of Exosomes in Oxidative Stress

Bovine milk-derived exosomes demonstrated significant potential to protect cells from oxidative stress, which has been confirmed in studies using the IEC-6 intestinal cell model [72]. Exosome treatment significantly increased the survival rate of the cells exposed to hydrogen peroxide (H_2_O_2_), a potent inducer of oxidative stress. Pretreatment of cells with exosomes for 48 h restored cell viability to 92.2% when compared to 77.8% in cells exposed to H_2_O_2_ alone. This protective effect was associated with a significant reduction (to near-control levels) in reactive oxygen species (ROS) levels, a 30–50% reduction in lipid peroxidation (as measured by malondialdehyde (MDA) levels), and lower lactate dehydrogenase (LDH) activity (even below control levels), which indicates the ability of exosomes to protect cell membrane integrity. Additionally, significant increased activity of key antioxidant enzymes, such as superoxide dismutase (SOD) and glutathione peroxidase (GPX), further underscored the role of exosomes in enhancing cellular antioxidant defenses. The results also demonstrated that exosomes increased the expression level of miR-146a (2.4-fold) and miR-155 (2.8-fold), which are known to regulate inflammatory response and oxidative stress [73,74]. Treatment of IEC-6 intestinal cells with exosomes reduced the H_2_O_2_-induced upregulation of *Nrf2* and *Ho1*, genes involved in the cellular defense against oxidative stress [75], to the levels observed in the control cells. A significant increase (by approximately 1.8-fold) in the level of HO-1 protein (which is crucial for cytoprotection under oxidative stress conditions [76]) was also observed in the experiment [72].

Further research by Wang et al. [77] showed that the administration of exosomes prior to the induction of oxidative stress with H_2_O_2_ reduced the production of ROS to near-control levels, improved cellular energy parameters, and reduced the levels of stress-related purine nucleotides such as AMP (by 24%) and GMP (by 27%). Energy status analysis showed that exosome treatment increased the total amount of adenylyl nucleotides and improved cellular energy charge. In addition, exosomes were found to modulate the activity of AMP-activated protein kinase, a key regulator of cellular energy metabolism [77]. The ratio of phosphorylated AMPK to total AMPK, which increased with H_2_O_2_ exposure, decreased by 18% after exosome treatment, which suggests that exosomes contribute to cellular adaptation to energy deficits [77]. These results suggest that bovine milk exosomes can act as a potent protective factor, supporting cell regeneration under oxidative stress conditions by modulating purine metabolism, energy status, and cell signaling.

### 7.2. Immunomodulatory Effects of Exosomes

In studies investigating the immunomodulatory properties of bovine milk-derived exosomes, experiments were conducted in healthy adult participants [78]. The subjects consumed one liter of milk, after which plasma levels of specific miRNAs (miR-15b, miR-21, miR-106b, and miR-223) were monitored at different time intervals. The analysis showed that miR-223 and miR-106b appeared in the plasma within 3 to 6 h after milk consumption. Notably, miR-223 and miR-106b exhibited 162% increase and 60% increase, receptively, in postprandial peak plasma concentration compared to baseline levels. Despite the increase in miRNA levels, milk consumption did not cause a direct increase in pro-inflammatory cytokines such as TNF-α, IL-1β, IL-6, or IL-10 in plasma [78]. The second phase of the study involved an in vitro analysis of the effects of bovine milk-derived exosomes on immune cells, i.e., peripheral blood mononuclear cells (PBMCs). PBMCs were isolated from participants before and after milk consumption and then stimulated with concanavalin A (ConA; a mitogen that activates T lymphocytes, leading to their proliferation and increased cytokine production [79]). The results demonstrated that PBMCs collected after milk consumption as well as those treated with miRNA-enriched exosomes secreted significantly higher levels of cytokines, including TNF-α (1.5-fold increase), IL-1β (1.7-fold increase), IL-6 (1.6-fold increase), and IL-10 (1.4-fold increase), compared to control cells [78]. These findings suggest that bovine milk-derived exosomes may modulate the immune response by enhancing the activity of immune cells when exposed to specific stimulating conditions.

### 7.3. Therapeutic Potential of Exosomes in Fibrosis

Bovine milk-derived exosomes have also shown promising therapeutic potential in the treatment of cardiac fibrosis. In the study by Zhang et al. [80], oral administration of exosomes (resuspended in PBS) to rats with isoproterenol-induced cardiac fibrosis led to a reduction in extracellular matrix deposition in the left ventricle. Western blot analysis revealed a significant decrease in the expression of fibrotic markers, including α-smooth muscle actin (42% reduction) and collagen type I (38% reduction), in the exosome-treated group compared to controls. Exosome-treated rats also showed improved cardiac function (increased ejection fraction by 19% and fractional shortening by 22% and decreased end-systolic volume by 28% and end-diastolic volume by 24%), as demonstrated by echocardiographic studies. The results of immunofluorescence analysis of heart tissue demonstrated an increase in the number of endothelial cells (by 34%) and increased VEGF expression (by 31%), suggesting that bovine milk-derived exosomes promote angiogenesis. In addition, the in vitro study on human umbilical vein endothelial cells (HUVECs) subjected to oxygen and glucose deprivation showed that exosomes stimulated proliferation (by 38%), migration (by 45%), and neovascularization (by 41%) compared to PBS-treated control cells [80]. The abovementioned findings suggest that oral administration of bovine milk-derived exosomes may alleviate cardiac fibrosis and support the regeneration of damaged tissue by promoting angiogenesis. As fibrosis is a challenging condition affecting various organs, including the lungs, liver, and kidneys [81], the efficacy of bovine milk-derived exosomes in alleviating cardiac fibrosis offers a potential therapeutic approach for fibrosis in other tissues. These results open new perspectives for fibrosis therapies, which currently have limited therapeutic options [81].

## 8. Potential Side Effects of Bovine Milk-Derived Exosomes

Although milk-derived exosomes hold great promise as therapeutic agents, their potential side effects must be carefully evaluated. These naturally occurring nanoparticles carry a diverse cargo, including proteins, lipids and nucleic acids such as DNA, mRNA, miRNA, and lncRNA [6]. While these molecules can provide therapeutic benefits, they also have the potential to interfere with unintended physiological pathways. For example, microRNAs within exosomes may inadvertently modulate gene expression in non-target cells, disrupting cellular homeostasis or leading to unintended adverse effects [82,83]. Exosomes can also influence immune responses through their biomolecular cargo, which can either overstimulate immune pathways or suppress critical immune functions. Such effects have been documented in studies with exosomes from other biological sources [83,84]. In addition, bovine milk-derived exosomes could theoretically contribute to tumor growth or progression, as has been observed with exosomes from other sources [84,85]. This potential arises from their ability to deliver biomolecules that promote cell proliferation or alter the tumor microenvironment. Similarly, exosomes may interact with infectious agents, potentially facilitating viral entry or replication by mimicking viral particles [83]. Another concern is the potential aggregation of exosomes in the body, particularly at high doses. Aggregation may accelerate immune clearance or lead to localized adverse effects, as demonstrated in preclinical studies [86]. Milk-derived exosomes represent a promising avenue for therapeutic development, but their potential side effects require careful investigation. Comprehensive preclinical and clinical studies are essential to assess their safety profile, particularly with regard to modulation of the immune system, long-term systemic effects, and interactions with pre-existing diseases or infections.

## 9. Challenges and Future Perspectives

Exosomes are natural carriers of bioactive molecules that can influence key biological processes, including the regulation of gene expression. Over the past two decades, there have been significant advances in the study of exosomes. The majority of research has focused on exosomes derived from human cells, as their endogenous origin and tissue compatibility make them ideal candidates for therapeutic applications [85,87]. More recently, exosomes derived from bovine milk have emerged as a promising area of investigation. The results of in vitro and in vivo studies suggest that bovine milk-derived exosomes could become a valuable tool in medical applications, particularly in the treatment of various diseases [88,89]. The fact that exosomes derived from bovine milk exhibit high biocompatibility and minimal systemic toxicity and are abundantly available, inexpensive to produce, and free from ethical concerns [88] positions them as an attractive alternative to human-derived exosomes. Nevertheless, the clinical application of bovine milk-derived exosomes in therapeutic strategies entails a range of scientific, technological, and regulatory challenges that must be addressed to realize their full potential.

One of the critical challenges in this field appears to be ensuring batch-to-batch consistency by standardizing the process of exosome isolation and purification. The natural origin of these nanoparticles, coupled with their heterogeneity and diversity of sources, makes it difficult to obtain consistent production batches [87,90]. Bovine milk-derived exosomes show significant variability in size, composition, and biological activity due to factors such as cow breed, feeding regimen, or farming conditions. Current methods, including ultracentrifugation and polymer precipitation, are time-consuming, expensive, and not very scalable [88,91,92]. Recent efforts have focused on implementing protocols to assess production reproducibility [93], but these approaches require further validation to ensure their reliability. Therefore, the development of robust methods to ensure consistent exosome composition and functionality is essential.

Another important aspect is quality control and the removal of potential contaminants. Bovine milk-derived exosomes may contain undesirable components such as host proteins or DNA residues that can induce undesired immune responses, posing a significant risk to clinical safety [87,92]. The implementation of rigorous quality-control procedures and the establishment of comprehensive regulatory standards are essential to mitigate these risks [87,94]. In addition, despite the low toxicity of bovine milk-derived exosomes, long-term preclinical studies are required to assess their safety and toxicity. Ensuring the stability of exosomes within the body is another critical challenge. Exogenous exosomes are rapidly cleared from circulation, significantly limiting their therapeutic efficacy [91,94]. It is therefore necessary to develop strategies to enhance the stability of exosomes and extend their half-life in systemic circulation.

Despite these challenges, bovine milk exosomes may present an opportunity in precision medicine, offering a cost-effective and scalable platform for the development of personalized therapies. With continued research and technological advancements, they could become a pivotal component in the future of therapeutic interventions. The current research, as reviewed in this paper, largely focuses on the therapeutic potential of bovine milk-derived exosomes, particularly in disease-related applications such as gut health, nervous system function, bone regeneration, skin repair, and hair growth. However, there are a number of directions for future research on exosomes. One promising avenue is their use in functional and medicinal foods and the development new dietary guidelines. In the future, bovine milk-derived exosomes may become an integral part of innovative nutritional solutions to improve human health.

## Data Availability

Not applicable.

## References

[1] Haug A., Høstmark A.T., Harstad O.M. (2007). Bovine Milk in Human Nutrition—A Review. Lipids Health Dis..

[2] Montgomery H., Haughey S.A., Elliott C.T. (2020). Recent food safety and fraud issues within the dairy supply chain (2015–2019). Glob. Food Secur..

[3] García-Martínez J., Pérez-Castillo Í.M., Salto R., López-Pedrosa J.M., Rueda R., Girón M.D. (2022). Beneficial Effects of Bovine Milk Exosomes in Metabolic Interorgan Cross-Talk. Nutrients.

[4] Rashidi M., Bijari S., Khazaei A.H., Shojaei-Ghahrizjani F., Rezakhani L. (2022). The Role of Milk-Derived Exosomes in the Treatment of Diseases. Front. Genet..

[5] Kalluri R., LeBleu V.S. (2020). The Biology, Function, and Biomedical Applications of Exosomes. Science.

[6] Gurunathan S., Kang M.H., Kim J.H. (2021). A Comprehensive Review on Factors Influencing Biogenesis, Functions, Therapeutic and Clinical Implications of Exosomes. Int. J. Nanomed..

[7] Kumar M.A., Baba S.K., Sadida H.Q., Marzooqi S.A., Jerobin J., Altemani F.H., Algehainy N., Alanazi M.A., Abou-Samra A.-B., Kumar R. (2024). Extracellular Vesicles as Tools and Targets in Therapy for Diseases. Signal Transduct. Target. Ther..

[8] Moghassemi S., Dadashzadeh A., Sousa M.J., Vlieghe H., Yang J., León-Félix C.M., Amorim C.A. (2024). Extracellular Vesicles in Nanomedicine and Regenerative Medicine: A Review over the Last Decade. Bioact. Mater..

[9] Shang R., Lee S., Senavirathne G., Lai E.C. (2023). MicroRNAs in Action: Biogenesis, Function and Regulation. Nat. Rev. Genet..

[10] Gurung S., Perocheau D., Touramanidou L., Baruteau J. (2021). The Exosome Journey: From Biogenesis to Uptake and Intracellular Signalling. Cell Commun. Signal..

[11] Lee Y.J., Shin K.J., Chae Y.C. (2024). Regulation of Cargo Selection in Exosome Biogenesis and Its Biomedical Applications in Cancer. Exp. Mol. Med..

[12] Abudoureyimu M., Zhou H., Zhi Y., Wang T., Feng B., Wang R., Chu X. (2019). Recent Progress in the Emerging Role of Exosome in Hepatocellular Carcinoma. Cell Prolif..

[13] Li Y., Xing L., Wang L., Liu X., Wu L., Ni M., Zhou Z., Li L., Liu X., Huang Y. (2023). Milk-Derived Exosomes as a Promising Vehicle for Oral Delivery of Hydrophilic Biomacromolecule Drugs. Asian J. Pharm. Sci..

[14] Mun D., Oh S., Kim Y. (2022). Perspectives on Bovine Milk-Derived Extracellular Vesicles for Therapeutic Applications in Gut Health. Food Sci. Anim. Resour..

[15] Li P., Kaslan M., Lee S.H., Yao J., Gao Z. (2017). Progress in exosome isolation techniques. Theranostics.

[16] Weiskirchen R., Schröder S.K., Weiskirchen S., Buhl E.M., Melnik B. (2023). Isolation of Bovine and Human Milk Extracellular Vesicles. Biomedicines.

[17] Yamauchi M., Shimizu K., Rahman M., Ishikawa H., Takase H., Ugawa S., Okada A., Inoshima Y. (2019). Efficient method for isolation of exosomes from raw bovine milk. Drug Dev. Ind. Pharm..

[18] Marsh S.R., Pridham K.J., Jourdan J., Gourdie R.G. (2021). Novel protocols for scalable production of high quality purified small extracellular vesicles from bovine milk. Nanotheranostics.

[19] Ko M., Kim H.J., Park J., Lee H., Lee K.N., Kim K., Lee J., Yoon S.J., Kim T., Jeong S. (2023). Isolation of bovine milk exosome using electrophoretic oscillation assisted tangential flow filtration with antifouling of micro-ultrafiltration membrane filters. ACS Appl. Mater. Interfaces.

[20] Kaddour H., Lyu Y., Shouman N., Mohan M., Okeoma C.M. (2020). Development of novel high-resolution size-guided turbidimetryenabled particle purification liquid chromatography (PPLC): Extracellular vesicles and membraneless condensates in focus. Int. J. Mol. Sci..

[21] Dilsiz N. (2024). A Comprehensive Review on Recent Advances in Exosome Isolation and Characterization: Toward Clinical Applications. Transl. Oncol..

[22] Wu D., Kittana H., Shu J., Kachman S.D., Cui J., Ramer-Tait A.E., Zempleni J. (2019). Dietary Depletion of Milk Exosomes and Their MicroRNA Cargos Elicits a Depletion of miR-200a-3p and Elevated Intestinal Inflammation and Chemokine (C-X-C Motif) Ligand 9 Expression in Mdr1a-/- Mice. Curr. Dev. Nutr..

[23] Peng S., Shen L., Yu X., Wu J., Zha L., Xia Y., Luo H. (2023). miR-200a Attenuated Oxidative Stress, Inflammation, and Apoptosis in Dextran Sulfate Sodium-Induced Colitis through Activation of Nrf2. Front. Immunol..

[24] Elia G., Guglielmi G. (2018). CXCL9 Chemokine in Ulcerative Colitis. Clin. Ter..

[25] Tong L., Hao H., Zhang Z., Lv Y., Liang X., Liu Q., Liu T., Gong P., Zhang L., Cao F. (2021). Milk-Derived Extracellular Vesicles Alleviate Ulcerative Colitis by Regulating the Gut Immunity and Reshaping the Gut Microbiota. Theranostics.

[26] Mun D., Kang M., Shin M., Choi H.J., Kang A.N., Ryu S., Unno T., Maburutse B.E., Oh S., Kim Y. (2023). Alleviation of DSS-Induced Colitis via Bovine Colostrum-Derived Extracellular Vesicles with MicroRNA Let-7a-5p Is Mediated by Regulating Akkermansia and β-Hydroxybutyrate in Gut Environments. Microbiol. Spectr..

[27] Nebenfuehr S., Kollmann K., Sexl V. (2020). The Role of CDK6 in Cancer. Int. J. Cancer.

[28] Mo C., Lou X., Xue J., Shi Z., Zhao Y., Wang F., Chen G. (2024). The Influence of Akkermansia muciniphila on Intestinal Barrier Function. Gut Pathog..

[29] Qi J., Gan L., Fang J., Zhang J., Yu X., Guo H., Cai D., Cui H., Gou L., Deng J. (2022). Beta-Hydroxybutyrate: A Dual Function Molecular and Immunological Barrier Function Regulator. Front. Immunol..

[30] Du C., Quan S., Zhao Y., Nan X., Chen R., Tang X., Xiong B. (2023). Bovine Milk-Derived Extracellular Vesicles Prevent Gut Inflammation by Regulating Lipid and Amino Acid Metabolism. Food Funct..

[31] DeClercq V., d’Eon B., McLeod R.S. (2015). Fatty Acids Increase Adiponectin Secretion through Both Classical and Exosome Pathways. Biochim. Biophys. Acta Mol. Cell Biol. Lipids.

[32] Holopainen M., Colas R.A., Valkonen S., Tigistu-Sahle F., Hyvärinen K., Mazzacuva F., Lehenkari P., Käkelä R., Dalli J., Kerkelä E. (2019). Polyunsaturated Fatty Acids Modify the Extracellular Vesicle Membranes and Increase the Production of Proresolving Lipid Mediators of Human Mesenchymal Stromal Cells. Biochim. Biophys. Acta Mol. Cell Biol. Lipids.

[33] Kominsky D.J., Campbell E.L., Colgan S.P. (2010). Metabolic Shifts in Immunity and Inflammation. J. Immunol..

[34] Maghraby M.K., Li B., Chi L., Ling C., Benmoussa A., Provost P., Postmus A.C., Abdi A., Pierro A., Bourdon C. (2021). Extracellular Vesicles Isolated from Milk Can Improve Gut Barrier Dysfunction Induced by Malnutrition. Sci. Rep..

[35] Li Y.Y., Xu Q.W., Xu P.Y., Li W.M. (2020). MSC-Derived Exosomal miR-34a/c-5p and miR-29b-3p Improve Intestinal Barrier Function by Targeting the Snail/Claudins Signaling Pathway. Life Sci..

[36] Tong L., Zhang S., Liu Q., Huang C., Hao H., Tan M.S., Yu X., Lou C.K.L., Huang R., Zhang Z. (2023). Milk-Derived Extracellular Vesicles Protect Intestinal Barrier Integrity in the Gut-Liver Axis. Sci. Adv..

[37] Groschwitz K.R., Hogan S.P. (2009). Intestinal Barrier Function: Molecular Regulation and Disease Pathogenesis. J. Allergy Clin. Immunol..

[38] Tong L., Hao H., Zhang X., Zhang Z., Lv Y., Zhang L., Yi H. (2020). Oral Administration of Bovine Milk-Derived Extracellular Vesicles Alters the Gut Microbiota and Enhances Intestinal Immunity in Mice. Mol. Nutr. Food Res..

[39] Zhu Y., Chen B., Zhang X., Akbar M.T., Wu T., Zhang Y., Zhi L., Shen Q. (2024). Exploration of the Muribaculaceae Family in the Gut Microbiota: Diversity, Metabolism, and Function. Nutrients.

[40] Mao J., Li S., Fu R., Wang Y., Meng J., Jin Y., Wu T., Zhang M. (2023). Sea Cucumber Hydrolysate Alleviates Immunosuppression and Gut Microbiota Imbalance Induced by Cyclophosphamide in Balb/c Mice through the NF-κB Pathway. Foods.

[41] Kushkevych I., Dordević D., Kollar P., Vítězová M., Drago L. (2019). Hydrogen Sulfide as a Toxic Product in the Small-Large Intestine Axis and its Role in IBD Development. J. Clin. Med..

[42] Liu Y., Yu X., Zhao J., Zhang H., Zhai Q., Chen W. (2020). The Role of MUC2 Mucin in Intestinal Homeostasis and the Impact of Dietary Components on MUC2 Expression. Int. J. Biol. Macromol..

[43] Waters R.A., Robinson J., Edwardson J.M. (2021). Syncollin Is an Antibacterial Polypeptide. Cell Microbiol..

[44] Saikh K.U. (2021). MyD88 and Beyond: A Perspective on MyD88-Targeted Therapeutic Approach for Modulation of Host Immunity. Immunol. Res..

[45] Du C., Quan S., Nan X., Zhao Y., Shi F., Luo Q., Xiong B. (2021). Effects of Oral Milk Extracellular Vesicles on the Gut Microbiome and Serum Metabolome in Mice. Food Funct..

[46] Zhang B., Zhao J., Jiang M., Peng D., Dou X., Song Y., Shi J. (2022). The Potential Role of Gut Microbial-Derived Exosomes in Metabolic-Associated Fatty Liver Disease: Implications for Treatment. Front. Immunol..

[47] Li T., Chen X., Qi Q., Feng X. (2024). Bovine Milk-Derived Exosomes Affect Gut Microbiota of DSS-Induced Colitis Mice. Indian J. Microbiol..

[48] Mutai E., Zhou F., Zempleni J. (2023). Depletion of Dietary Bovine Milk Exosomes Impairs Sensorimotor Gating and Spatial Learning in C57BL/6 Mice. J. Dairy Sci..

[49] Zhou F., Ebea P., Mutai E., Wang H., Sukreet S., Navazesh S., Dogan H., Li W., Cui J., Ji P. (2022). Small Extracellular Vesicles in Milk Cross the Blood-Brain Barrier in Murine Cerebral Cortex Endothelial Cells and Promote Dendritic Complexity in the Hippocampus and Brain Function in C57BL/6J Mice. Front. Nutr..

[50] O’Brien J., Hayder H., Zayed Y., Peng C. (2018). Overview of MicroRNA Biogenesis, Mechanisms of Actions, and Circulation. Front. Endocrinol..

[51] Föger-Samwald U., Dovjak P., Azizi-Semrad U., Kerschan-Schindl K., Pietschmann P. (2020). Osteoporosis: Pathophysiology and Therapeutic Options. EXCLI J..

[52] Oliveira M.C., Di Ceglie I., Arntz O.J., van den Berg W.B., van den Hoogen F.H., Ferreira A.V., van Lent P.L., van de Loo F.A. (2017). Milk-Derived Nanoparticle Fraction Promotes the Formation of Small Osteoclasts but Reduces Bone Resorption. J. Cell Physiol..

[53] Go G., Jeon J., Lee G., Lee J.H., Lee S.H. (2021). Bovine Milk Extracellular Vesicles Induce the Proliferation and Differentiation of Osteoblasts and Promote Osteogenesis in Rats. J. Food Biochem..

[54] Yun B., Maburutse B.E., Kang M., Park M.R., Park D.J., Kim Y., Oh S. (2020). Dietary Bovine Milk-Derived Exosomes Improve Bone Health in an Osteoporosis-Induced Mouse Model. J. Dairy Sci..

[55] Hao H., Liu Q., Zheng T., Li J., Zhang T., Yao Y., Liu Y., Lin K., Liu T., Gong P. (2024). Oral Milk-Derived Extracellular Vesicles Inhibit Osteoclastogenesis and Ameliorate Bone Loss in Ovariectomized Mice by Improving Gut Microbiota. J. Agric. Food Chem..

[56] Huang S.-C., He Y.-F., Chen P., Liu K.-L., Shaukat A. (2023). Gut microbiota as a target in the bone health of livestock and poultry: Roles of short-chain fatty acids. Anim. Dis..

[57] Oliveira M.C., Pieters B.C.H., Guimarães P.B., Duffles L.F., Heredia J.E., Silveira A.L.M., Oliveira A.C.C., Teixeira M.M., Ferreira A.V.M., Silva T.A. (2020). Bovine Milk Extracellular Vesicles Are Osteoprotective by Increasing Osteocyte Numbers and Targeting RANKL/OPG System in Experimental Models of Bone Loss. Front. Bioeng. Biotechnol..

[58] Mobley C., Mumford P.W., McCarthy J.J., Miller M.E., Young K.C., Martin J.S., Beck D.T., Lockwood C.M., Roberts M.D. (2017). Whey Protein-Derived Exosomes Increase Protein Synthesis and Hypertrophy in C2-C12 Myotubes. J. Dairy Sci..

[59] Leiferman A., Shu J., Grove R., Cui J., Adamec J., Zempleni J. (2018). A Diet Defined by Its Content of Bovine Milk Exosomes and Their RNA Cargos Has Moderate Effects on Gene Expression, Amino Acid Profiles and Grip Strength in Skeletal Muscle in C57BL/6 Mice. J. Nutr. Biochem..

[60] Parry H.A., Mobley C.B., Mumford P.W., Romero M.A., Haun C.T., Zhang Y., Roberson P.A., Zempleni J., Ferrando A.A., Vechetti I.J. (2019). Bovine Milk Extracellular Vesicles (EVs) Modification Elicits Skeletal Muscle Growth in Rats. Front. Physiol..

[61] Han G., Kim H., Kim D.E., Ahn Y., Kim J., Jang Y.J., Kim K., Yang Y., Kim S.H. (2022). The Potential of Bovine Colostrum-Derived Exosomes to Repair Aged and Damaged Skin Cells. Pharmaceutics.

[62] Bai R., Guo Y., Liu W., Song Y., Yu Z., Ma X. (2023). The Roles of WNT Signaling Pathways in Skin Development and Mechanical-Stretch-Induced Skin Regeneration. Biomolecules.

[63] Cabral-Pacheco G.A., Garza-Veloz I., Castruita-De la Rosa C., Ramirez-Acuña J.M., Perez-Romero B.A., Guerrero-Rodriguez J.F., Martinez-Avila N., Martinez-Fierro M.L. (2020). The Roles of Matrix Metalloproteinases and Their Inhibitors in Human Diseases. Int. J. Mol. Sci..

[64] Lu L., Bai W., Wang M., Han C., Du H., Wang N., Gao M., Li D., Dong F., Ge X. (2024). Novel Roles of Bovine Milk-Derived Exosomes in Skin Antiaging. J. Cosmet. Dermatol..

[65] Kim Y., Lim K.M. (2021). Skin barrier dysfunction and filaggrin. Arch. Pharm. Res..

[66] Ni C., Zhang Z., Wang Y., Zhang Z., Guo X., Lv H. (2023). Hyaluronic acid and HA-modified cationic liposomes for promoting skin penetration and retention. J. Control. Release.

[67] Jang Y.N., Lee J.O., Lee J.M., Park A.Y., Kim Y.J., Kim S.Y., Seok J., Yoo K.H., Kim B.J. (2024). Exosomes derived from human dermal fibroblasts (HDFn-Ex) alleviate DNCB-induced atopic dermatitis (AD) via PPARα. Exp. Dermatol..

[68] Bae I.S., Kim S.H. (2021). Milk Exosome-Derived MicroRNA-2478 Suppresses Melanogenesis through the Akt-GSK3β Pathway. Cells.

[69] Jaśkiewicz A., Pająk B., Orzechowski A. (2018). The Many Faces of Rap1 GTPase. Int. J. Mol. Sci..

[70] Kim H., Jang Y., Kim E.H., Jang H., Cho H., Han G., Song H.K., Kim S.H., Yang Y. (2022). Potential of Colostrum-Derived Exosomes for Promoting Hair Regeneration Through the Transition from Telogen to Anagen Phase. Front. Cell Dev. Biol..

[71] Hu S., Li Z., Lutz H., Huang K., Su T., Cores J., Dinh P.C., Cheng K. (2020). Dermal exosomes containing miR-218-5p promote hair regeneration by regulating β-catenin signaling. Sci. Adv..

[72] Wang L., Shi Z., Wang X., Mu S., Xu X., Shen L., Li P. (2021). Protective Effects of Bovine Milk Exosomes Against Oxidative Stress in IEC-6 Cells. Eur. J. Nutr..

[73] Gilyazova I., Asadullina D., Kagirova E., Sikka R., Mustafin A., Ivanova E., Bakhtiyarova K., Gilyazova G., Gupta S., Khusnutdinova E. (2023). MiRNA-146a-A Key Player in Immunity and Diseases. Int. J. Mol. Sci..

[74] Mahesh G., Biswas R. (2019). MicroRNA-155: A Master Regulator of Inflammation. J. Interferon Cytokine Res..

[75] Loboda A., Damulewicz M., Pyza E., Jozkowicz A., Dulak J. (2016). Role of Nrf2/HO-1 system in development, oxidative stress response and diseases: An evolutionarily conserved mechanism. Cell Mol. Life Sci..

[76] Chiang S.K., Chen S.E., Chang L.C. (2021). The Role of HO-1 and Its Crosstalk with Oxidative Stress in Cancer Cell Survival. Cells.

[77] Wang L., Wang X., Shi Z., Shen L., Zhang J., Zhang J. (2021). Bovine Milk Exosomes Attenuate the Alteration of Purine Metabolism and Energy Status in IEC-6 Cells Induced by Hydrogen Peroxide. Food Chem..

[78] Mutai E., Ramer-Tait A.E., Zempleni J. (2020). MicroRNAs in Bovine Milk Exosomes Are Bioavailable in Humans but Do Not Elicit a Robust Pro-Inflammatory Cytokine Response. ExRNA.

[79] Lai Y.-C., Chuang Y.-C., Chang C.-P., Yeh T.-M. (2015). Macrophage Migration Inhibitory Factor Has a Permissive Role in Concanavalin A-Induced Cell Death of Human Hepatoma Cells Through Autophagy. Cell Death Dis..

[80] Zhang C., Lu X., Hu J., Li P., Yan J., Ling X., Xiao J. (2022). Bovine Milk Exosomes Alleviate Cardiac Fibrosis via Enhancing Angiogenesis In Vivo and In Vitro. J. Cardiovasc. Transl. Res..

[81] Brigstock D.R. (2021). Extracellular Vesicles in Organ Fibrosis: Mechanisms, Therapies, and Diagnostics. Cells.

[82] Tzng E., Bayardo N., Yang P.C. (2023). Current challenges surrounding exosome treatments. Extracell. Vesicle.

[83] Dal’Forno-Dini T., Birck M.S., Rocha M., Bagatin E. (2024). Exploring the reality of exosomes in dermatology. An. Bras. Dermatol..

[84] Dilsiz N. (2021). Hallmarks of exosomes. Future Sci. OA.

[85] Rezaie J., Feghhi M., Etemadi T. (2022). A Review on Exosomes Application in Clinical Trials: Perspective, Questions, and Challenges. Cell Commun. Signal..

[86] Ranjan P., Colin K., Dutta R.K., Verma S.K. (2023). Challenges and Future Scope of Exosomes in the Treatment of Cardiovascular Diseases. J. Physiol..

[87] Butreddy A., Kommineni N., Dudhipala N. (2021). Exosomes as Naturally Occurring Vehicles for Delivery of Biopharmaceuticals: Insights from Drug Delivery to Clinical Perspectives. Nanomaterials.

[88] Adriano B., Cotto N.M., Chauhan N., Jaggi M., Chauhan S.C., Yallapu M.M. (2021). Milk Exosomes: Nature’s Abundant Nanoplatform for Theranostic Applications. Bioact. Mater..

[89] Prasadani M., Kodithuwakku S., Pennarossa G., Fazeli A., Brevini T.A.L. (2024). Therapeutic Potential of Bovine Milk-Derived Extracellular Vesicles. Int. J. Mol. Sci..

[90] Ghodasara A., Raza A., Wolfram J., Salomon C., Popat A. (2023). Clinical Translation of Extracellular Vesicles. Adv. Healthc. Mater..

[91] Koh H.B., Kim H.J., Kang S.W., Yoo T.H. (2023). Exosome-Based Drug Delivery: Translation from Bench to Clinic. Pharmaceutics.

[92] Sharma A., Yadav A., Nandy A., Ghatak S. (2024). Insight into the Functional Dynamics and Challenges of Exosomes in Pharmaceutical Innovation and Precision Medicine. Pharmaceutics.

[93] Korchak J.A., Wiest E.F., Zubair A.C. (2023). How Do We Assess Batch-to-Batch Consistency Between Extracellular Vesicle Products?. Transfusion.

[94] Chavda V.P., Pandya A., Kumar L., Raval N., Vora L.K., Pulakkat S., Patravale V., Salwa S., Duo Y., Tang B.Z. (2023). Exosome Nanovesicles: A Potential Carrier for Therapeutic Delivery. Nano Today.

